# Alanine aminotransferase/aspartate aminotransferase ratio is the best surrogate marker for insulin resistance in non-obese Japanese adults

**DOI:** 10.1186/1475-2840-11-117

**Published:** 2012-10-01

**Authors:** Ryuichi Kawamoto, Katsuhiko Kohara, Tomo Kusunoki, Yasuharu Tabara, Masanori Abe, Tetsuro Miki

**Affiliations:** 1 Department of Community Medicine, Ehime University Graduate School of Medicine, Toon-city, Ehime, Japan; 2 Department of Geriatric Medicine, Ehime University Graduate School of Medicine, Toon-city, Ehime, Japan; 3 Center for Genomic Medicine, Kyoto University Graduate School of Medicine, Sakyo-ku, Kyoto, 606-8501, Japan; 4 Department of Internal Medicine, Seiyo Municipal Nomura Hospital, Seiyo-city, Ehime, 797-1212, Japan

**Keywords:** ALT/AST ratio, Insulin resistance, Marker, Body mass index

## Abstract

**Background:**

The aim of the present study was to examine how liver markers are associated with insulin resistance in Japanese community-dwelling adults.

**Methods:**

This cross-sectional study included 587 men aged 58 ± 14 (mean ± standard deviation; range, 20–89) years and 755 women aged 60 ± 12 (range, 21–88) years. The study sample consisted of 998 (74.4%) non-obese [body mass index (BMI) <25.0 kg/m^2^] and 344 (25.6%) overweight (BMI ≥25 kg/m^2^) subjects. Insulin resistance was defined by homeostasis model assessment of insulin resistance (HOMA-IR) of at least 2.5, and HOMA-IR and potential confounders were compared between the groups. Areas under the curve (AUC) of the receiver operating characteristic curves (ROC) were used to compare the power of these serum markers.

**Results:**

In non-obese subjects, the best marker of insulin resistance was alanine aminotransferase (ALT)/aspartate aminotransferase (AST) ratio of 0.70 (95% confidence interval (CI), 0.63-0.77). In overweight subjects, AUC values for the ALT/AST ratio and ALT were 0.66 (0.59-0.72) and 0.66 (0.59-0.72), respectively. Multiple linear regression analyses for HOMA-IR showed that ALT/AST ratios were independently and significantly associated with HOMA-IR as well as other confounding factors in both non-obese and overweight subjects. The optimal cut-off point to identifying insulin resistance for these markers yielded the following values: ALT/AST ratio of ≥0.82 in non-obese subjects and ≥1.02 in overweight subjects. In non-obese subjects, the positive likelihood ratio was greatest for ALT/AST ratio.

**Conclusions:**

In non-obese Japanese adults, ALT/AST ratio may be the best reliable marker of insulin resistance.

## Background

Obesity is also a major worldwide public health problem and is associated with a high risk of developing insulin resistance [1]. Insulin resistance plays an important role in the pathogenesis of incident diabetes, hypertension, dyslipidemia, and cardiovascular disease (CVD) [2-4]. Detailed measurement of insulin resistance requires the use of diffuse techniques (e.g., glucose clamp technique.) that require expense and time. Alternatives have been sought to simplify the measurement of insulin resistance and one is Homeostatic Model Assessment of insulin resistance (HOMA-IR), which uses fasting insulin and glucose levels to calculate insulin resistance [5] and correlates reasonably with the results of clamping studies. The use of this index is problematic, however, in that insulin levels are not measured during the usual annual health examination and in clinical practice.

Many studies have demonstrated that alanine aminotransferase (ALT), aspartate aminotransferase (AST) and gamma-glutamyl transferase (GGT) levels independently predict type 2 diabetes [6-10], metabolic syndrome [11-14], and CVD [8]. These markers have been shown to be associated with indirect measurements of insulin resistance including fasting insulin levels [14] and HOMA-IR [10,15-17]. However, in Japanese community-dwelling persons, there are few studies to demonstrate a relationship between liver markers and insulin resistance, categorized by BMI. It is important for us to be able to evaluate insulin resistance by measuring the liver markers which are inexpensive and routinely measured in clinical setting.

We took advantage of the large representative sample of Japanese adults who participated at the time of their annual health examination. We investigated how liver markers were associated with insulin resistance in healthy Japanese adults. For this, we used cross-sectional data from community-dwelling participants without clinical diabetes.

## Methods

### Subjects

The present study is designed as a part of the Nomura study [18]. Subjects were selected through a community-based annual check-up process in a rural town located in Ehime prefecture, Japan. A sample of 3,164 subjects was recruited and the available sample population compromised 2,764 subjects. Information on medical history, present conditions, and drugs was obtained by interview. Other characteristics, such as smoking and alcohol habits, and medication, were investigated by individual interviews using a structured questionnaire. Subjects taking medications for hypertension (N = 736), diabetes (N = 153), or dyslipidemia (N = 108) were excluded (N = 877). For all these individuals, overnight fasting plasma samples were available for measuring immunoreactive insulin (IRI) and high sensitivity C-reactive protein (hsCRP). We reduced the number of subjects (N = 545) because the omitted subjects’ samples had a limited volume of plasma. The final study sample included 1,342 eligible persons. The final study sample included 587 men and 755 women. This study was approved by the ethics committee of Ehime University School of Medicine, and written informed consent was obtained from each subject.

### Evaluation of confounding factors

Information on demographic characteristics and risk factors was collected using clinical files. Body mass index was calculated by dividing weight (in kilograms) by the square of the height (in meters). Smoking status was defined as the number of cigarette packs per day multiplied by the number of years smoked (pack·year), and the participants were classified into never smokers, past smokers, light smokers (<30 pack·year) and heavy smokers (≥30 pack;year). The daily alcohol consumption was measured using the Japanese liquor unit in which a unit corresponds to 22.9 g of ethanol, and the participants were classified into never drinkers, occasional drinkers (<1 unit/day), light drinkers (1–1.9 unit/day), and heavy drinkers (≥2 unit/day). We measured blood pressure in the right upper arm of the participants in a sedentary position using an automatic oscillometric blood pressure recorder (BP-103i; Colin, Aichi, Japan) while the subjects were seated after having rested for at least 5 min. Total cholesterol (T-C), triglycerides (TG), high-density lipoprotein cholesterol (HDL-C), fasting plasma glucose (FPG), uric acid, hsCRP, high molecular weight (HMW) adiponectin (FUJIREBIO, Tokyo, Japan), GGT, ALT, AST, and IRI were measured during fasting. Low-density lipoprotein cholesterol (LDL-C) levels were calculated using the Friedewald formula [19]. Participants with TG levels ≥400 mg/dl were excluded. HOMA-IR was calculated from FPG and IRI levels using the following formula; [FPG (mg/dL) X IRI (mU/mL)]/405 [5], and a level of Insulin resistance was defined as HOMA-IR ≥2.5 [5].

### Statistical analysis

Statistical analysis was performed using IBM SPSS Statistics Version 20 (Statistical Package for Social Science Japan, Inc., Tokyo, Japan). All values are expressed as mean ± standard deviation (SD), unless otherwise specified. Data for TG, hsCRP, serum HMW adiponectin, GGT, ALT, and AST were skewed, and are presented as median (interquartile range) values, and were log-transformed for analysis. Subjects were divided into two groups based on BMI [non-obese, <25.0 kg/m^2^; overweight, ≥25.0 kg/m^2^ (waist circumference was not available in this study)], and differences between the two groups were determined by Student’s t-test and χ^2^ test. In addition, areas under the receiver operating characteristic (ROC) curves were determined for each variable to identify the predictors of insulin resistance. Areas under the ROC curves are provided with standard errors. An ROC curve is a plot of the sensitivity (true positive) versus 1–specificity (false positive) for each potential marker tested. The area under the ROC curve is a summary of the overall diagnostic accuracy of the test. The best markers have ROC curves that are shifted to the left with areas under the curve near unity. Nondiagnostic markers are represented by diagonals with areas under the ROC curves close to 0.5. Likelihood ratios were calculated as the ratios of sensitivity/(1-specificity) (positive likelihood ratio) and (1-sensitivity)/specificity (negative likelihood ratio). Multiple linear regression analysis was used to evaluate the contribution of each confounding factor for HOMA-IR. A value of *P* <0.05 was considered significant.

## Results

### Background factors of subjects categorized by BMI

Table 1 shows the value of each background factor categorized by BMI. The subjects comprised 587 men aged 58 ± 14 (range, 20–89) years and 755 women aged 60 ± 12 (range, 21–88) years. The mean BMI in the study sample was 23.1 ± 3.1 kg/m^2^, with 998 (74.4%) non-obese (BMI <25.0 kg/m^2^) and 344 (25.6%) overweight (BMI ≥25 kg/m^2^). Prevalence of smoker, systolic blood pressure (SBP), diastolic blood pressure (DBP), T-C, TG, LDL-C, uric acid, hsCRP, GGT, ALT, AST, and ALT/AST ratio were significantly higher in subjects with a BMI ≥25.0 kg/m^2^, but age, HDL-C and serum HMW adiponectin were significantly lower in that group. There were no inter-group differences in sex, alcohol consumption, and prevalence of CVD.

**Table 1:** **Characteristics of subjects categorizedby body mass index**

**Body mass index† characteristics**	**Total**	**Non-obese**	**Overweight**	*P*-value*
	**All N = 1,342**	**<25.0 kg/m**^**2**^** N = 998**	**≥25.0 kg/m**^**2**^** N = 344**	
Male sex,%	43.7	42.8	46.5	0.232
Age (years)	59 ± 13	60 ± 13	57 ± 12	0.012
Body mass index (kg/m^2^)	23.1 ± 3.1	21.7 ± 2.0	27.1 ± 2.2	<0.001
Smoking status {never/ex/light/heavy (%)}	71.2/9.6/9.1/10.1	72.6/8.3/8.9/10.1	66.9/13.4/9.6/10.2	0.043
Alcohol consumption {never/light/moderate/heavy (%)}	38.8/31.8/18.6/10.8	40.5/30.5/18.9/10.1	34.0/35.8/17.4/12.8	0.072
Cardiovascular disease,%	4.5	4.7	4.1	0.764
Systolic blood pressure (mmHg)	134 ± 21	132 ± 21	139 ± 19	<0.001
Diastolic blood pressure (mmHg)	80 ± 11	79 ± 11	84 ± 10	<0.001
Total cholesterol (mg/dL)	201 ± 35	200 ± 34	205 ± 35	0.024
Triglycerides (mg/dL)	90 (67–126)	85 (64–117)	108 (81–152)	<0.001
HDL cholesterol (mg/dL)	63 ± 15	65 ± 16	57 ± 13	<0.001
LDL cholesterol (mg/dL)	118 ± 32	116 ± 31	123 ± 32	<0.001
Uric acid (mg/dL)	5.0 ± 1.4	4.8 ± 1.4	5.5 ± 1.4	<0.001
High sensitivity CRP (mg/dL)	0.04 (0.02-0.09)	0.04 (0.02-0.07)	0.07 (0.04-0.15)	<0.001
Serum HMW adiponectin (μg/mL)	4.89 (2.98-7.99)	5.37 (3.21-8.69)	3.95 (2.24-5.82)	<0.001
GGT (IU/L)	25 (18–43)	22 [17-37]	32 (21–63)	<0.001
ALT (IU/L)	17 [13-24]	16 [12-21]	22 [16-31]	<0.001
AST (IU/L)	21 [18-26]	21 [18-26]	22 [19-27]	0.013
ALT/AST ratio	0.79 (0.65-1.0)	0.74 (0.63-0.91)	0.96 (0.72-1.2)	<0.001

HDL, high-density lipoprotein; LDL, low-density lipoprotein; CRP; C-reactive protein; HMW, high molecular weight; GGT, Gamma-glutamyl transferase; ALT, alanine aminotransferase; AST, aspartate aminotransferase. Data presented are mean ± standard deviation. Data for triglycerides, high sensitivity CRP, serum HMW adiponectin, GGT, ALT, AST, and ALT/AST ratio were skewed, and are presented as median (interquartile range), and were log-transformed for analysis. * Student’s t-test or χ^2^ test.

### Insulin resistance of subjects categorized by BMI

FBG, IRI, and HOMA-IR were significantly higher in overweight subjects (Table 2), and prevalence of insulin resistance (HOMA-IR ≥1.6 or ≥2.5) was significantly higher in overweight subjects than in non-obese subjects.

**Table 2:** **Insulin resistance of subjectscategorized by body massindex**

**Body mass index characteristics**	**Total**	**Non-obese**	**Overweight**	*P*-value*
	**All N = 1,342**	**<25.0 kg/m**^**2**^** N = 998**	**≥25.0 kg/m**^**2**^** N = 344**	
Fasting blood glucose (mg/dL)	92 (87–99)	91 (86–98)	96 (89–103)	<0.001
Immunoreactive insulin (mU/mL)	5.2 (3.4-7.7)	4.5 (3.0-6.6)	8.0 (5.4-11.2)	<0.001
HOMA-IR†	1.20 (0.76-1.84)	1.03 (0.67-1.54)	1.93 (1.23-2.81)	<0.001
HOMA-IR† <1.6,%	67.5	77.5	38.7	
HOMA-IR† ≥1.6 and <2.5,%	20.0	16.3	30.8	<0.001
HOMA-IR† ≥2.5,%	12.4	6.2	30.5	<0.001

HOMA-IR, homeostasis of minimal assessment of insulin resistance. Data for fasting blood glucose, fasting immunoreactive insulin, and HOMA-IR were skewed, and are presented as median (interquartile range). †HOMA-IR was calculated using the following formula; [fasting blood glucose (FBG) (mg/dL) X fasting Immunoreactive insulin (mU/mL)]/405. Data for fasting blood glucose, Immunoreactive insulin, and HOMA-IR were log-transformed for analysis. *Student’s t test or χ^2^ test.

### Comparison of areas under ROC curves [95% confidence interval] for potential markers of insulin resistance of subjects categorized by BMI

**Figure 1:**
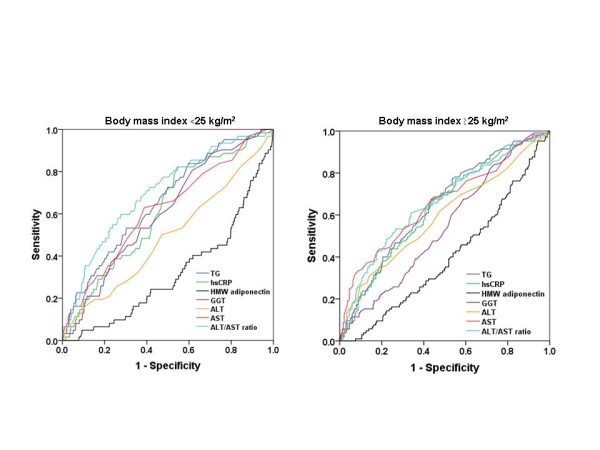
**Receiver operating characteristics (ROC)curves.** Sensitivity represents the true-positive results and 1-specificity, the false-positive results. The best markers have ROC curves that are shifted to the left with areas under the curve near unity. Nondiagnostic markers are represented by diagonals with areas under the ROC curves close to 0.5.

In non-obese subjects, the ROC curve analyses showed that the best marker of insulin resistance was ALT/AST ratio, with an area under the ROC curve of 0.70 (0.63-0.77) (Table 3; Figure 1). Serum HMW adiponectin, ALT, and hsCRP also discriminated insulin resistance, as they had areas under the ROC curve of 0.32 (0.25-0.39), 0.62 (0.55-0.69), and 0.61 (0.55-0.68), respectively. In overweight subjects, ALT/AST ratio and ALT were more effective.

**Table 3:** Comparison of areas under the ROC curves (95% CI) for potential markers of insulin resistance (HOMA-IR ≥2.5) of subjects categorized by body mass index

**Body mass index characteristics**	**AUC (95% CI)**					
	**Total**	***P*-value**	**Non-obese**	***P*-value**	**Overweight**	***P*-value**
	**All N = 1,342**		**<25.0 kg/m**^**2**^** N = 998**		**≥25.0 kg/m**^**2**^** N = 344**	
Triglycerides (mg/dL)	0.69 (0.65-0.73)	<0.001	0.66 (0.59-0.73)	<0.001	0.65 (0.58-0.71)	<0.001
High sensitivity CRP (mg/dL)	0.68 (0.64-0.72)	<0.001	0.61 (0.55-0.68)	0.003	0.63 (0.57-0.70)	<0.001
Serum HMW adiponectin (μg/mL)	0.32 (0.28-0.36)	<0.001	0.32 (0.25-0.39)	<0.001	0.40 (0.33-0.46)	0.003
GGT (IU/L)	0.63 (0.58-0.67)	<0.001	0.61 (0.54-0.67)	0.005	0.54 (0.48-0.61)	0.211
ALT (IU/L)	0.69 (0.64-0.73)	<0.001	0.62 (0.55-0.69)	0.002	0.66 (0.59-0.72)	<0.001
AST (IU/L)	0.56 (0.51-0.61)	0.013	0.49 (0.41-0.57)	0.818	0.59 (0.52-0.66)	0.007
ALT/AST ratio	0.74 (0.70-0.78)	<0.001	0.70 (0.63-0.77)	<0.001	0.66 (0.59-0.72)	<0.001

ROC, receiver operating characteristics; CI, confidence interval; AUC, area under ROC curve. Data for triglycerides, high sensitivity CRP, serum HMW adiponectin, GGT, ALT, AST, and ALT/AST ratio were skewed and log-transformed for analysis.

### Relationship between various confounding factors and HOMA-I of subjects categorized by body mass index

**Figure 2:**
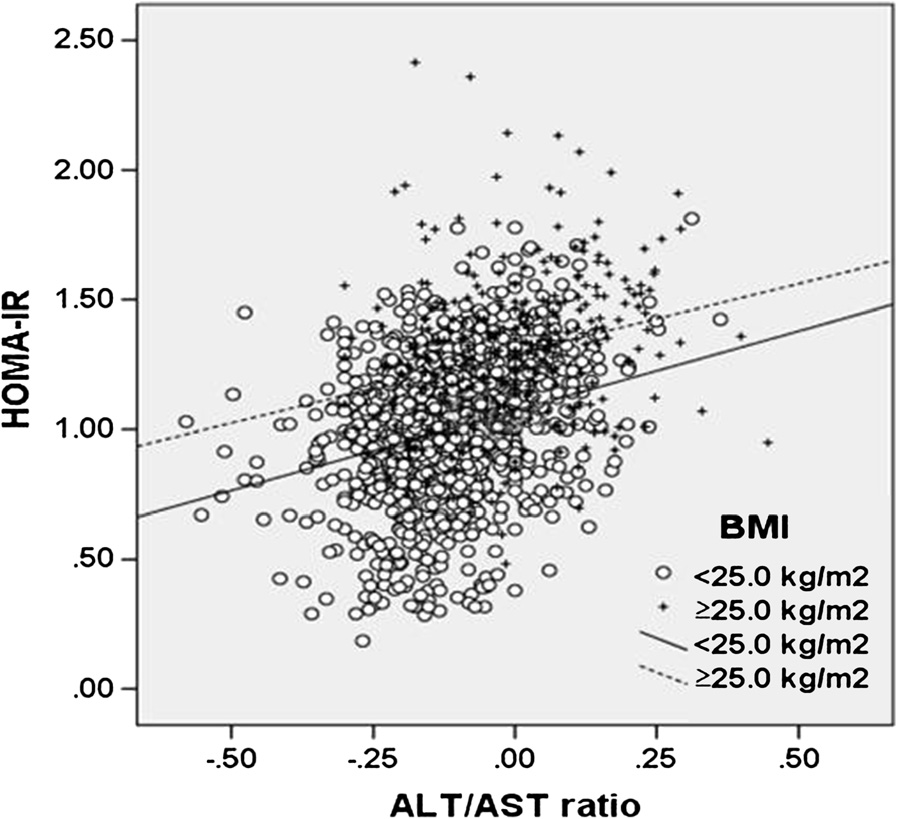
**Correlation between AST/ALT ratio and HOMA-IR categorized by BMI.** Data for HOMA-IR were skewed and log-transformed for analysis. The lines of best fit (BMI <25.0 kg/m^2^: r = 0.274, *P* < 0.001; BMI ≥25.0 kg/m^2^: r = 0.253, *P* < 0.001) are indicated.

Figure 2 shows the relationships between AST/ALT ratio and HOMA-IR. AST/ALT ratio was also significantly associated with measures of HOMA-IR in both non-obese (r = 0.274, *P* < 0.001) and overweight subjects (r = 0.253, *P* < 0.001). To further investigate whether AST/ALT ratio can explain the changes in HOMA-IR levels independent of other known confounding factors, multiple linear regression analyses for HOMA-IR showed that AST/ALT ratio was independently and significantly associated with HOMA-IR in both non-obese (β = 0.129, *P* < 0.001) and overweight subjects (β = 0.143, *P* = 0.038) (Table 4).

**Table 4:** Multiple linear regression analysis of the correlation between various confounding factors and HOMA-IR of subjects categorized by body mass index

**Body mass index† characteristics**	**β(*P*-value)**		
	**Total**	**Non-obese**	**Overweight**
	**All N = 1,342**	**<25.0 kg/m**^**2**^** N = 998**	**≥25.0 kg/m**^**2**^** N = 344**
Male sex,%	0.247 (<0.001)	0.281 (<0.001)	0.219 (0.005)
Age (years)	−0.064 (0.020)	−0.116 (0.001)	0.094 (0.135)
Body mass index (kg/m^2^)	0.320 (<0.001)	0.206 (<0.001)	0.262 (<0.001)
Smoking status {never/ex/light/heavy (%)}	−0.065 (0.014)	−0.060 (0.075)	−0.103 (0.073)
Alcohol consumption {never/light/moderate/heavy(%)}	−0.114 (<0.001)	−0.137 (<0.001)	−0.081 (0.222)
Cardiovascular disease,%	0.032 (0.140)	0.055 (0.046)	−0.024 (0.610)
Systolic blood pressure (mmHg)	0.093 (<0.001)	0.126 (<0.001)	0.032 (0.543)
Triglycerides (mg/dL)	0.138 (<0.001)	0.145 (<0.001)	0.164 (0.005)
HDL cholesterol (mg/dL)	−0.033 (0.215)	−0.038 (0.249)	−0.028 (0.645)
LDL cholesterol (mg/dL)	0.041 (0.079)	0.063 (0.034)	0.010 (0.848)
Uric acid (mg/dL)	0.021 (0.451)	0.049 (0.153)	−0.052 (0.400)
High sensitivity CRP (mg/dL)	0.049 (0.035)	0.048 (0.095)	0.074 (0.149)
Serum HMW adiponectin (μg/mL)	−0.164 (<0.001)	−0.162 (<0.001)	−0.231 (0.001)
GGT (IU/L)	0.097 (0.001)	0.079 (0.031)	0.149 (0.038)
ALT/AST ratio	0.122 (<0.001)	0.129 (<0.001)	0.143 (0.038)
R^2^	0.417 (<0.001)	0.315 (<0.001)	0.290 (<0.001)

Data for triglycerides, high sensitivity CRP, serum HMW adiponectin, GGT, ALT/AST ratio and HOMA-IR were skewed and log-transformed for analysis.

### Optimal cut-off point of ALT/AST ratio for predicting insulin resistance of subjects categorized by BMI

Table 5 shows the cut-off points of ALT/AST ratio for identifying insulin resistance. The optimal cut-off point to identifying insulin resistance for these markers yielded the following values: ALT/AST ratio of ≥0.82 in non-obese subjects, and ≥1.02 in overweight. In non-obese subjects, the positive likelihood ratio value indicates that the odds of insulin resistance increased by 1.91-fold if ALT/AST ratio was positive (the value ≥0.82). The negative likelihood ratios indicate the extent to which the odds of insulin resistance decrease if the test is negative. These odds also decreased more so for ALT/AST ratio. In overweight subjects, these values were similar.

**Table 5:** Comparison of ALT/AST ratio for predicting of insulin resistance (HOMA-IR ≥2.5) of subjects categorized by body mass index

**Characteristics cut-off point**	**HOMA-IR§**						
	**<2.5**	**≥2.5**	**Sensitivity**	**Specificity**	**Positive LR**	**Negative LR**	**Accuracy**
Body mass index <25.0 kg/m^2^	N = 936	N = 62					%
ALT/AST ratio <0.82	612	22	0.67	0.65	1.91	0.51	65.3
ALT/AST ratio ≥0.82	324	40					
Body mass index ≥25.0 kg/m^2^	N = 239	N = 105					
ALT/AST ratio <1.02	158	41	0.66	0.61	1.69	0.56	64.5
ALT/AST ratio ≥1.02	81	64					

LR, likelihood ratio.

### Relationship between ALT/AST ratio and insulin resistance of subjects within selected subgroups

Next, to control potential confounding by gender and BMI, the data were further stratified by gender and BMI (Table 6). In both genders with a BMI of 22.0 to 25.0 kg/m^2^, AST/ALT ratio was a reliable marker of insulin resistance, but in subjects with a BMI of <22.0 kg/m^2^, AST/ALT ratio was not significantly associated with HOMA-IR.

**Table 6:** Association between ALT/AST ratio and insulin resistance (HOMA-IR ≥2.5) and HOMA-IR within selected subgroups

**Stratified subgroups**	**N**	**HOMA-IR ≥2.5,%**	**ALT/AST ratio**		
			**ROC curves AUC (95%CI)**	**BMI-adjusted β (*P*-value)**	**Multivariate-adjusted β (*P*-value)**
Men Body mass index (kg/m^2^)					
Total	587	66 (11.2)	0.75 (0.68-0.81	0.253 (<0.001)	0.104 (0.016)
<22.0	202	7 (3.5)	0.67 (0.44-0.89)	0.257 (<0.001)	0.141 (0.051)
22.0-25.0	225	19 (8.4)	0.64 (0.51-0.77)	0.317 (<0.001)	0.166 (0.030)
<25.0	427	26 (6.1)	0.67 (0.56-0.77)	0.274 (<0.001)	0.143 (0.004)
≥25.0	160	40 (25.0)	0.73 (0.63-0.82)	0.215 (0.005)	0.052 (0.600)
Women Body mass index (kg/m^2^)					
Total	755	101 (13.4)	0.75 (0.70-0.81)	0.197 (<0.001)	0.104 (0.002)
<22.0	308	10 (3.2)	0.74 (0.62-0.87)	0.149 (0.008)	0.055 (0.334)
22.0-25.0	263	26 (9.9)	0.71 (0.60-0.82)	0.250 (<0.001)	0.136 (0.039)
<25.0	571	36 (6.3)	0.74 (0.65-0.82)	0.194 (<0.001)	0.092 (0.026)
≥25.0	184	65 (35.3)	0.68 (0.60-0.76)	0.231 (0.001)	0.176 (0.019)

Multivariate-adjusted for confounding factors in Table 4.

## Discussion

In the present study, we examined whether liver markers (e.g., GGT, ALT, AST, and ALT/AST ratio) as well as gender, age, BMI, smoking status, alcohol consumption, SBP, lipid profiles, uric acid, and serum HMW adiponectin were associated with insulin resistance in Japanese adults, categorized by body mass index. Most fundamental is the fact that not all overweight or obese persons are insulin resistant. In non-obese subjects, 6.2% of them were insulin resistant, and the best marker of insulin resistance was ALT/AST ratio, but serum HMW adiponectin, ALT, and hsCRP also discriminated insulin resistance. In the overweight subjects, areas of the ALT/AST ratio and ALT were greater than those of the other parameters. The optimal cut-off point to identifying insulin resistance for these markers yielded the following values: ALT/AST ratio of ≥0.82 in the non-obese, and ≥1.02 in the overweight subjects. The positive likelihood ratio was greatest for ALT/AST ratio in the non-obese subjects. ALT/AST ratio, an inexpensive and routinely measured clinical variable, might be used as an integrated parameter and measure to evaluate insulin resistance in community-dwelling persons, especially non-obese subjects.

Insulin resistance is common and when clustered with glucose intolerance, dyslipidemia and high blood pressure, and present in type 2 diabetes it play a key role in the occurrence of hyperglycemia. Resistance to insulin-mediated glucose disposal is distributed continuously through the general population [20], and we have no criterion with which to identify a participant as being insulin resistance. However, we classified a participant with a HOMA-IR of >2.5 as insulin resistance [21]. Some previous studies have demonstrated that HOMA-IR strongly correlates with glucose clamp–assessed insulin resistance [5,20] and has the advantage of requiring only a single plasma sample assayed for insulin and glucose. However, HOMA-IR is less accurate and precise than the glucose clamp method in measuring insulin resistance, but this limitation is now mitigated, because the HOMA model has become a widely used clinical and epidemiological tool, as in our study [22,23]. Thus, we used HOMA-IR as a marker of insulin resistance in this study.

In previous studies, several lipid ratios have been proposed as simple and useful clinical indicators of insulin resistance. The TG/HDL-C, the T-C/HDL-C, and the LDL-C/HDL-C ratio have shown similar potential for insulin resistance, though the reports are not entirely consistent [24-27]. Also in our study, both LDL-C/HDL-C and TG/HDL-C ratio were useful markers of insulin resistance, especially in all subjects or non-obese subjects. However, these markers were weaker in overweight subjects [28]. ALT/AST ratio as well as LDL-C/HDL-C and the TG/HDL-C ratio were also strongly related to insulin resistance. Several studies have reported a significant association of ALT with HOMA-IR [15,29,30]. Moreover, Hanley et al. [30,31] reported that ALT/AST ratio predicts metabolic syndrome independently of potential confounding variables, including directly measured insulin sensitivity and acute insulin response. ALT/AST ratio that includes information on at least two measures might have a more integrated explanation than single measures such as ALT or TG.

There are a number of possible mechanisms that can explain the association between the ALT/AST ratio and insulin resistance. Fat accumulation in the liver is characterized by several features of insulin resistance in normal weight and moderately overweight subjects, independent of BMI and intra-abdominal and overall obesity [32]. Increased liver fat content, a disorder that has detrimental effects on components of the metabolic syndrome, is known to be significantly correlated with these markers [33]. Nanji et al. [34] reported a significant correlation between ALT/AST ratio and the degree of fatty infiltration of the liver. Moreover, non-alcoholic fatty liver disease (NAFLD), which has recently been proposed as a feature of the metabolic syndrome [35], was also characterized by chronic elevations in liver transaminase levels, including ALT, AST, and GGT [36,37]. ALT/AST ratio and ALT per se along with the cut-off points might be reflecting NAFLD, but in this study, we have not evaluated ultrasound liver findings. Although, this study is of interest because liver transaminase markers, which are inexpensive and routinely collected in clinical settings, may provide a simple and accurate enhancement to models currently used to identify subjects with insulin resistance.

Some limitations of this study must be considered. First, the cross-sectional study design is limited in its ability to eliminate causal relationships between ALT/AST ratio and HOMA-IR. Second, our definition of HOMA-IR is based on a single assessment of FBS and IRI, which may introduce misclassification bias, although common to most epidemiological studies. However, random errors due to the fluctuations of laboratory measurements usually lead to a reduced estimate of the associated strength. Third, our study participants might include patients with subclinical liver diseases (i.e., chronic viral hepatitis, or drug–induced liver injury). These liver diseases are present in community-dwelling persons and are usually asymptomatic. Therefore the demographics and referral source may limit generalizability.

## Conclusions

The present study demonstrated that ALT/AST ratio is associated with insulin resistance according to BMI in a general population. The ability to identify who is non-obese or overweight and who is insulin resistant could help health care professionals in bringing about lifestyle interventions. In that context, use of the cutoff-points of ALT/AST ratio described in this report is simple and useful. The present data documented that in non-obese Japanese adults ALT/AST ratio may be the best reliable marker of insulin resistance. Further prospective population-based studies are needed to investigate the changes in lipid metabolism by lifestyle interventions.

## Competing interests

The authors declare that they have no competing interests.

## Authors’ contributions

RK, YT, and KK participated in the design of the study, performed the statistical analysis and drafted the manuscript. TK and AM contributed to acquisition of data and its interpretation. RK and MA contributed to conception and design of the statistical analysis. TM conceived of the study, participated in its design, coordination and helped to draft the manuscript. All authors read and approved the manuscript.
